# Trends in Availability of Genetic Tests in the United States, 2012–2022

**DOI:** 10.3390/jpm13040638

**Published:** 2023-04-06

**Authors:** Alyssa L. Halbisen, Christine Y. Lu

**Affiliations:** 1 Harvard Medical School, Harvard Pilgrim Health Care Institute, Boston, MA 02215, USA; 2 Kolling Institute, Faculty of Medicine and Health, The University of Sydney, The Northern Sydney Local Health District, Sydney, NSW 2077, Australia; 3 School of Pharmacy, Faculty of Medicine and Health, The University of Sydney, Sydney, NSW 2006, Australia

**Keywords:** genetic testing, genetic testing registry, genetic test availability

## Abstract

Globally, genetic testing has become increasingly used over the last two decades. As a result of the rapid development of genetic tests, the Genetic Testing Registry was created in the United States to provide transparent information on genetic tests and the corresponding laboratories. Using publicly available data from the Genetic Testing Registry, we analyzed trends in the availability of genetic tests in the United States over the last decade. As of November 2022, a total of 129,624 and 197,779 genetic tests in the US and globally, respectively, including updated versions of previously existing tests, have been made available and submitted to the genetic testing registry. Over 90% of tests submitted to GTR are for clinical rather than research purposes. Worldwide, 1081 and 6214 new genetic tests had been made available in 2012 and in 2022, respectively. In 2012, only 607 and in 2022, 3097 new genetic tests were made available in the US, with 2016 seeing the biggest increase in availability of new tests during the study period. Over 90% of tests can be used for diagnosis. In the US, 10 of >250 laboratories account for 81% of new genetic tests in GTR. As more genetic tests become available, further international collaboration is required for a comprehensive understanding of the available genetic tests worldwide.

## 1. Introduction

Since the completion of the Human Genome Project, the use and development of genetic testing has dramatically increased [1]. Precision medicine has transformed over the last two decades to support disease diagnosis and screening, predict disease risk, inform patient drug responsiveness, and understand individual ancestry [2,3,4] by accounting for individual variability in genes and other lifestyle factors [1,5]. Genetic testing data are being used in conjunction with clinical, familial, and demographic data to shape the diagnosis and treatment of disease, and institutions globally are implementing precision medicine initiatives to further invest in our understanding of disease [5,6]. Much like the rest of the world, the United States has seen a rapid expansion in genetic testing over the last decade.

In response to the growth in both number and complexity of genetic tests, the Genetic Testing Registry (GTR) was created in 2008 by recommendation from the United States’ Secretary’s Advisory Committee on Genetics, Health, and Society (SACGHS) [7]. GTR was developed to increase access to detailed information about each existing genetic test, standardized laboratory, and test-specific information, and to help health care providers make informed decisions about test orders [7].

GTR was developed by the National Center for Biotechnology Information (NCBI) under the guidance of the National Institutes of Health (NIH) and is maintained through a collaborative effort between federal agencies, health care providers, researchers, testing laboratories, advisory groups, insurance companies, and other key stakeholders [7,8]. Submission to GTR is optional but recommended. In the US, only laboratories certified by the Centers for Medicare and Medicaid Services (CMS) are permitted to submit genetic test data. CMS regulates the release of tests for the diagnosis, prevention, or treatment of any disease or impairment of, or the assessment of the health through the Clinical Laboratory Improvement Amendments of 1988 (CLIA) [9,10]. To enhance analytical validity, laboratories performing genetic testing must be CLIA certified or CLIA exempt in order to submit genetic testing information to GTR.

The objective of this study was to describe the availability of genetic tests in the United States from 2012–2022 through publicly available, downloadable data and through the interactive platform on the GTR website [11].

## 2. Materials and Methods

To accurately examine new and existing tests in the US, we used both publicly available downloadable data and GTR’s interactive webpage [11].

Genetic test submission to the GTR is broken down into three categories: minimal, recommended, and optional (Supplementary Materials). All laboratories submitting genetic test information to GTR must complete all minimally required components including test tracking ID, GTR accession number (if editing or deleting a previous entry), laboratory test name, purpose of the test, test performance location, method category, test method, analytical validity, condition/phenotype, whether a test is germline or somatic, target category, name of what gene(s) is tested, and indication type (if novel condition) [12]. Genetic tests registered to GTR can be flagged for one or more of the following test purposes: diagnosis, drug response, recurrence, monitoring, predictive, presymptomatic, prognostic, risk assessment, screening, and/or therapeutic management. Diagnostic tests aid in the indication or confirmation of disease, often when symptoms are present. Presymptomatic and predictive tests are performed on asymptomatic individuals at risk for developing a disease. Predictive tests can be used to determine the treatment response relative to cancer biomarkers. Similar to predictive tests, prognostic tests are applicable to cancer biomarkers and can predict the aggressiveness and overall outcome of the disease at the time of diagnosis. Screening tests are often used among patients in a target population affected by a genetic condition or by those who have the potential to transmit disease to their offspring. Screening tests also include newborn screening. Recurrence tests determine disease recurrence in patients who have been diagnosed with cancer and have been treated. Drug response tests are pharmacogenetic tests that evaluate a patient’s response to medications. Therapeutic management tests are used to determine therapeutic decision making [11,12]. Test service and specimen type are also optionally indicated on the interactive database for each test. This includes whether the test is for “custom mutation-specific/carrier testing” or “custom prenatal testing” [11,12]. For this analysis, we did not differentiate tests by specimen collection type or test service type. New test submissions and edits to existing test were updated weekly.

### 2.1. Interactive Database

First, we gathered a baseline understanding of the current genetic test availability worldwide. The GTR website illustrates the current landscape of global genetic tests by offering an interactive database to filter and search for registered tests. To begin, we calculated the total number of genetic tests globally and their relative categories. We then filtered tests by country to generate the total number of current clinical and research tests in the US. We then limited the results to only clinical tests and filtered the database by laboratories to generate the number of laboratories who perform genetic testing. From this filter, we were able to gather the number of tests related to each test purpose category. Finally, we compared the results from the interactive database to those from the downloadable dataset.

### 2.2. Downloadable Data

We then downloaded a publicly available, comprehensive dataset including all currently existing genetic tests on the registry [13]. First, we stratified the dataset by US and non-US tests because our analysis largely focused on the growth of test availability in the United States. We then calculated the annual number of current tests in the US and indicated whether they were used clinically or for research. Current test status and test type were flagged in the dataset by GTR.

GTR accepts genetic test submissions worldwide and indicates where each genetic test is developed. Once published on the registry, genetic tests are given a unique identifying number in the format “GTR000000000.x”. The number after the decimal relates to the test version, as tests are edited and updated over time. We then limited the dataset to include only first versions of each test to create a time trend analysis of new genetic tests in the United States.

We were also interested in understanding the number of laboratories creating new genetic tests. Using the laboratory’s corresponding CLIA number, we calculated the number of new and current tests developed by each laboratory. CLIA certification relates to the type of testing performed at each laboratory. To receive CLIA certification, genetic tests must be evaluated for analytic validity; clinical validity; clinical utility; and for ethical, legal, and social implications [6]. Laboratories located in Washington State and New York State are exempt from CLIA certification due to statewide licensure that complies CLIA certification regulations. Laboratories that only perform waived tests determined by the Food and Drug Administration for posing an insignificant risk of erroneous results are also exempt from CLIA accreditation [6,9].

Finally, we linked the summary dataset with each genetic test purpose category to examine the number of newly introduced and current tests per purpose category. We then separated the tests by clinical or research test type to further understand the use of current genetic tests. Because some tests can be used for multiple purposes, the test purpose categories are not mutually exclusive. However, the purpose categories provide insight into how genetic testing may be used.

## 3. Results

### 3.1. Interactive Database

As of November 2022, there were 76,326 current genetic tests on GTR worldwide., where 76,083 (99.7%) tests were clinical and 243 (0.3%) were for research purposes. Of the 76,326 current tests, 37,289 (48.91%) belonged to laboratories in the US; 37,124 (99.56%) and 165 (0.44%) tests were for clinical and research purposes, respectively.

In the US, 200 laboratories performed 37,124 current clinical tests on the interactive database; 29 laboratories performed whole exome sequencing (WES) and 17 laboratories performed whole genome sequencing (WGS).

**Figure 1:**
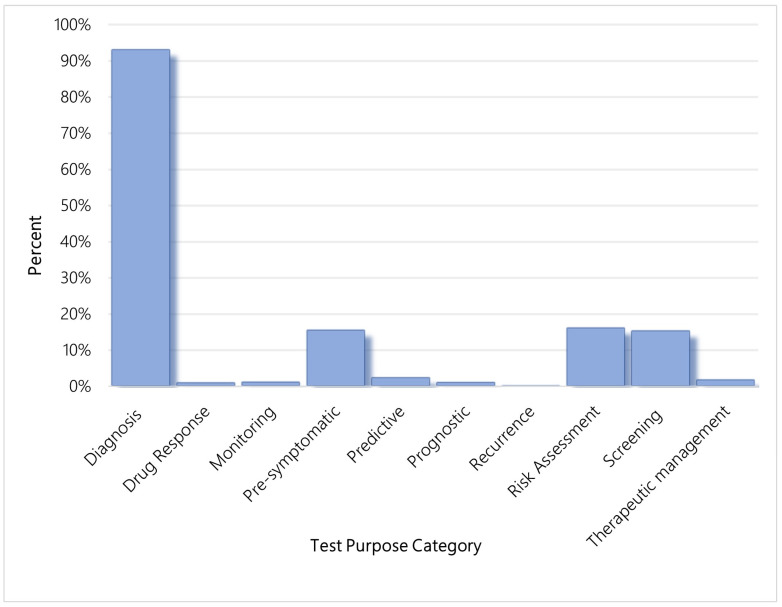
Percent of current genetic tests within each test purpose category, US.

Figure 1 represents the percent of current genetic tests within each test purpose category. Because genetic tests are often multipurpose, test purpose categories are not mutually exclusive.

### 3.2. Downloadable Data

Worldwide, a total of 197,779 genetic tests were developed and submitted to the genetic testing registry as of November 2022, including updated versions of previously existing tests. In the US, a total of 129,624 genetic tests were developed and submitted to the genetic testing registry as of November 2022, including updated versions of previously existing tests. Genetic tests in the US account for about 66% of all genetic tests and subsequent versions on GTR.

**Figure 2:**
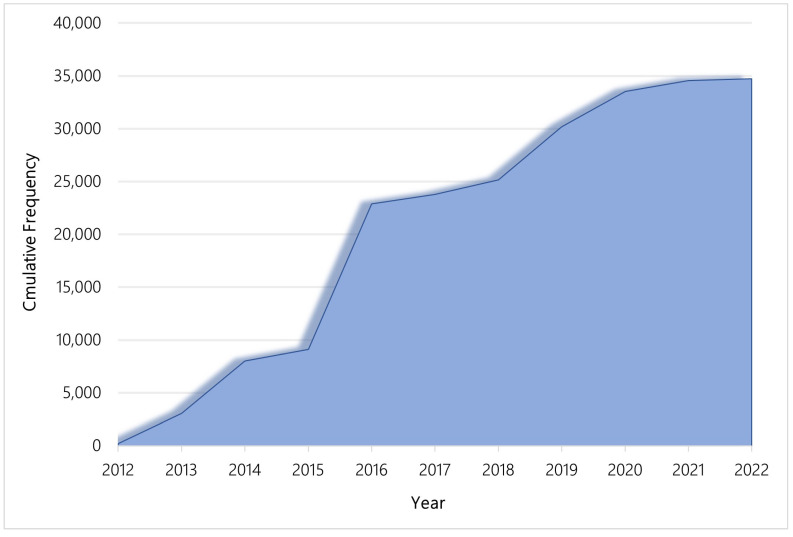
Cumulative frequency of current genetic tests per year, US.

Each year, laboratories improve many of their existing tests and discontinue older versions. As of November 2022, there were 76,302 and 37,271 current genetic tests worldwide and in the US, respectively. Figure 2 represents the cumulative number of current genetic tests in the US from 197 tests in 2012 to 37,271 tests in 2022. In the US, 37,106 (99.55%) were for clinical purposes and only 165 (0.45%) were for research purposes, as recorded in GTR.

**Figure 3:**
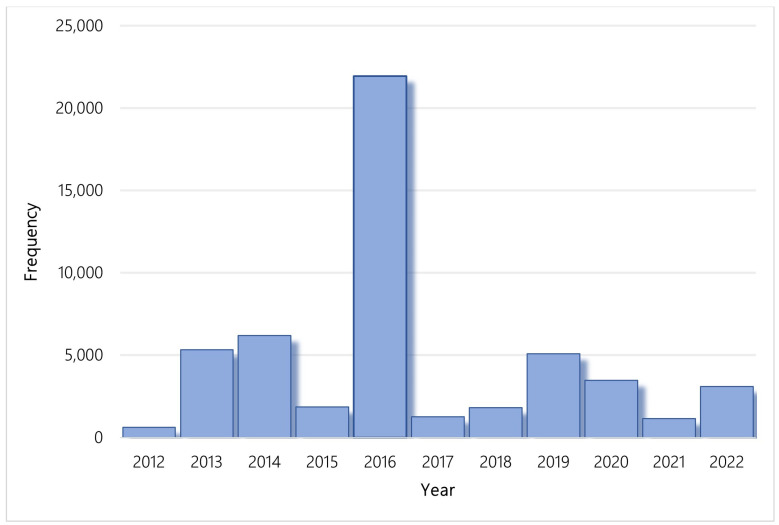
Number of new genetic tests per year, US.

Worldwide, 1081 new genetic tests were made available in 2012 and, cumulatively, there were a total of 106,007 new genetic tests introduced and registered in GTR between January 2012 and November 2022. Over the past 10 years, a total of 51,803 new genetic tests were made available in the US, an increase from the 607 new genetic tests in 2012. In the publicly available datasets, test type is not indicated for every iteration of test. Of 51,803 new genetic tests, 13,315 had a clinical or research indication. Of those tests, 13,276 (99.7%) were clinical and 39 (0.3%) were for research. Figure 3 illustrates the annual number of new genetic tests in the United States from 2012 to 2022.

**Figure 4:**
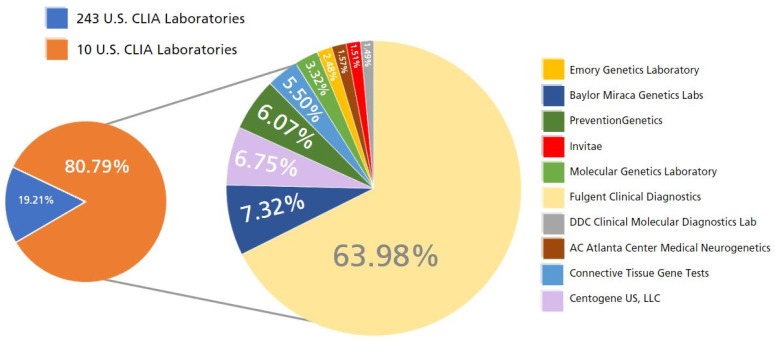
Percentage of new genetic tests in the U.S. from 2012 to 2022 by CLIA-certified laboratory.

Figure 4 represents the percentage of new genetic tests created by corresponding Clinical Laboratory Improvement Amendments (CLIA) certified laborites in the United States. Of the 51,803 new genetic tests introduced to the market in the US between 2012 and 2022, 51,574 (99.56%) were from CLIA certified laboratories and 229 were from non-CLIA certified laboratories.

Of the 51,803 new genetic tests, 92.93% of these (47,929 tests) were developed by 31 CLIA-certified laboratories and 80.79% (41,666 tests) were developed by only 10 CLIA-certified laboratories across the US.

**Figure 5:**
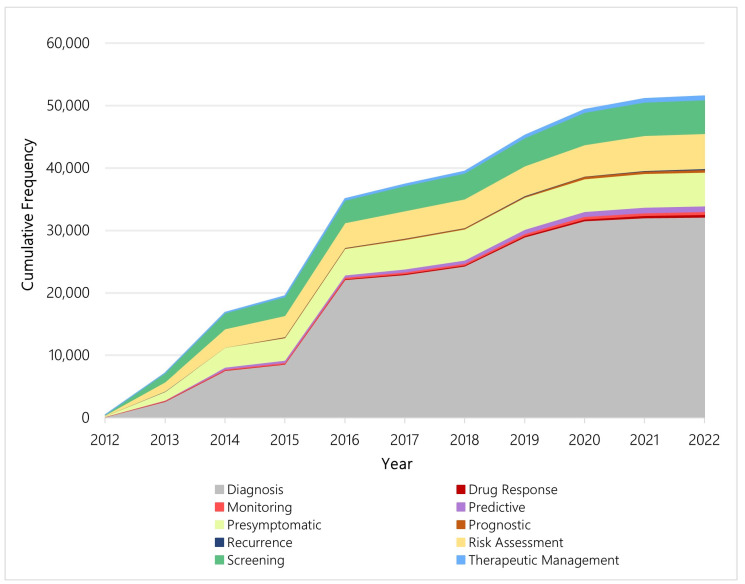
Cumulative frequency of new genetic tests per year by test purpose category, US.

From 2012 to 2022, 32,185 (62.13%) new genetic tests registered to the GTR in the US are diagnostic. Diagnosis is the largest genetic test purpose category on the registry. After diagnosis, 5646 (10.9%) new genetic tests are for risk assessment, 5410 (10.4%) new genetic tests are for pre-symptomatic testing, and 5370 (10.4%) are for screening. Figure 5 illustrates the cumulative increase in genetic tests by test purpose category in the US across the study period.

**Figure 6:**
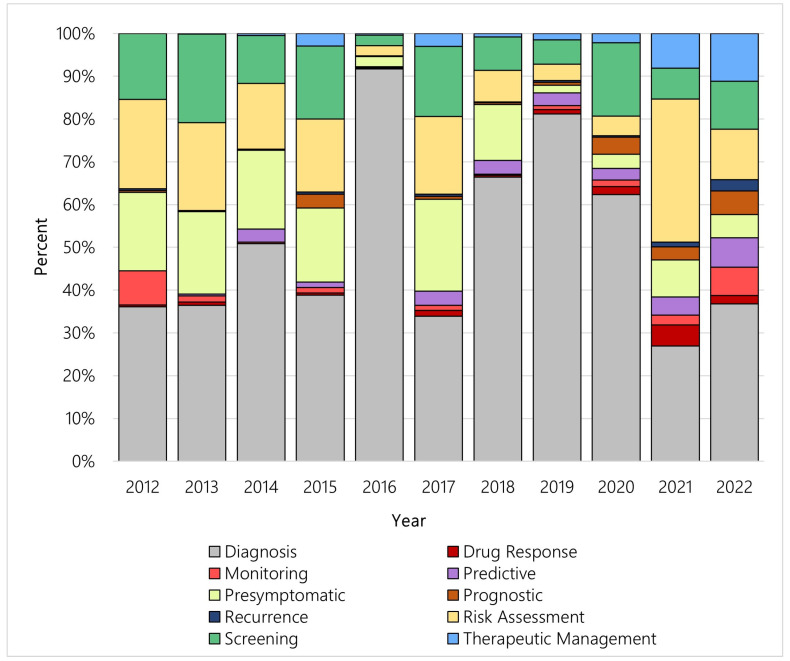
Corresponding category of new genetic tests introduced per year, US.

**Figure 7:**
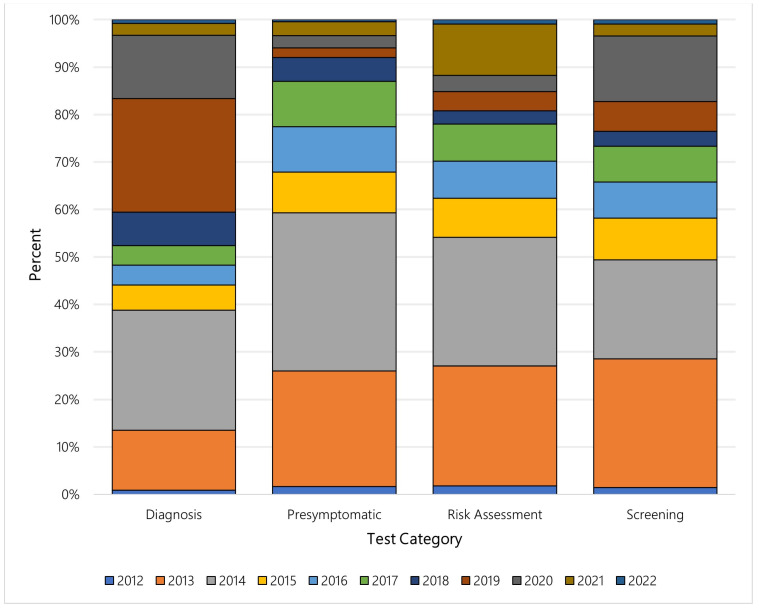
Annual distribution of new genetic tests among the most common test purpose categories, US.

Each year, new tests for different purposes are introduced, and Figure 6 illustrates the percent of new genetic tests by category from 2012 to 2022 in the US. Tests for diagnosis dominated each year over the study period, particularly 2016 and 2019. Within the categories, however, the distribution of newly available genetic tests shifted over time. Among the four most common test purpose categories—diagnosis, pre-symptomatic, risk assessment, and screening—more than half of all tests related to pre-symptomatic testing, risk assessment testing, or screening were made available between 2012 and 2015. In contrast, most tests related to diagnosis were made available after 2015. Figure 7 illustrates the yearly shift among the four aforementioned test purpose categories.

## 4. Discussion

This is a longitudinal study describing the trends in newly available and current genetic tests in the United States from 2012 to 2022. The number of new genetic tests increased from 607 tests in 2012 to 51,803 tests in 2022. The largest increase in new genetic tests was between 2015 and 2016 when 21,952 genetic tests were developed and added to GTR. Of the 21,952 new tests introduced in 2016, 19,561 were from Fulgent Clinical Diagnostics and their associated labs. Fulgent Clinical Diagnostics is one of 10 labs performing over 80% of all genetic tests in the US.

We found a significant shift in genetic test purpose in the US throughout our study period. Among all pre-symptomatic tests, 66.97% were introduced between 2012–2015. Similarly for tests for risk assessment and for screening, more than half were made available between 2012–2015 (60.65% and 55.84%, respectively). Less than half of these tests were made available in more recent years: 33.03%, 39.35%, and 44.16% of pre-symptomatic tests, risk assessment tests, and screening, respectively, between 2016 and 2022. In contrast, of all the diagnostic tests, only 26.63% were made available between 2012 and 2015 compared with 73.37% between 2016 and 2022. This trend mirrors the global trend in overall diagnostic testing. Because of external factors such as an ageing population and the increased prevalence of chronic and infectious disease, in conjunction with scientific advances in diagnostic testing, the global diagnostic test industry is expected to have a compound annual growth rate of 8.63% by 2030 [14]. The overall global diagnostic (genetic and non-genetic) testing market, currently valued at $158.6 billion (USD) in 2021, is expected to surpass $348.8 billion (USD) in 2030 [15]. Furthermore, the diagnostic testing market in the US is expected to reach $40.1 billion (USD), or 25% of the global market value, by 2026 [15].

The increase in genetic test availability and use in the US is not wavering. In January 2015, President Barack Obama launched a Precision Medicine Initiative investing $215 million dollars (USD) to support research, development, and innovation of precision medicine [16]. The same year, 20-year patents on BRCA 1/2 genes by Myriad Genetics expired—two years after the Supreme Court’s decision in the landmark Association for Molecular Pathology v. Myriad Genetics ruled that human genes cannot be patented. This decision opened the doors for other labs to develop testing for specific genes, ultimately resulting in more accessible and affordable genetic tests [17].

One strength of this study is very little research has been conducted using the genetic testing registry database that captures test information from the US and worldwide. Our analysis provided critical insight into the patterns of genetic test availability in the last decade and identified that the development and availability of diagnostic tests dominated the global market in recent years. Future research using this publicly available data can provide insight into possible gaps in test availability. In contrast, there are some limitations worth discussing. Our results may underestimate the total counts of available genetic tests given the voluntary nature of the database, and very likely underestimate available genetic tests in other countries. Nevertheless, genetic testing availability has undoubtedly exploded in the last decade, even if our analysis likely underestimated the magnitude of this growth because of the incomplete capture of tests by the GTR database.

In the US, only specified federal agencies considered “regulatory” can require organizations to provide information and adhere to policies set forth by the agency. The National Institutes of Health (NIH) is not a regulatory agency and thus cannot require laboratories to submit genetic test data. When a laboratory does choose to submit genetic test data, they are not required to complete all portions of the submission application. Each application is separated into three parts—minimal, recommended, and optional. Laboratories are only required to fill out the minimally required information, which may result in incomplete data.

Moreover, the GTR database is not confirmed by the NIH. Test submitters are required to adhere to a code of conduct regulated by the NIH, and the NIH has incorporated quality assurance measures to ensure accurate information. Despite these measures, however, genetic test data may be inaccurate or incomplete. Finally, although the database provides health care providers and patients with a tool to better understand the available genetic tests, its data are ultimately incomplete given its voluntary nature. This analysis likely underestimates the magnitude in which genetic tests exist in the US, but undoubtedly genetic testing availability has exploded in the last decade. Mandatory reporting from labs and greater international collaboration are needed for a better understanding of the genetic testing availability in the US and globally, and for identifying gaps in testing development.

## 5. Conclusions

An increasing number and range of genetic tests have been made available in the US and worldwide between 2012 and 2022, based on our analysis of the Genetic Testing Registry, a voluntary database created by the NIH. The US accounts for over 60% of the tests registered on GTR. Most of the tests registered on GTR are for clinical purposes. Over 90% of available genetic tests can be used for diagnosis, with huge increases in such tests since 2016. In the US, 10 of >250 laboratories account for 81% of new tests in GTR. As more genetic tests become available, required test reporting to GTR is needed for a clear understanding of the genetic testing landscape. Further international collaboration is required for a greater understanding of the available genetic tests worldwide.

## Data Availability

Data available in a publicly accessible repository. The data presented in this study are openly available on the Genetic Testing Registry database; https://ftp.ncbi.nlm.nih.gov/pub/GTR/data/_README.html, accessed on 23 November 2022.

## Supplementary Materials

The following supporting information can be downloaded at: https://www.mdpi.com/article/10.3390/jpm13040638/s1, Supplementary Document: Required, Recommended, and Optional components of GTR’s genetic test submission document.
