# Supplementary material for: Trends in Availability of Genetic Tests in the United States, 2012–2022
Source: J Pers Med. 2023 Apr 6;13(4):638. doi: 10.3390/jpm13040638 (PMC10142561; doi:10.3390/jpm13040638)
Supplement: Supplementary file 1 [file jpm-13-00638-s001.zip › jpm-2121222-supplementary.pdf]

Laboratories submitting genetic test information to the Genetic Testing Registry must fill out a submission form containing minimal (required), recommended, and optional information about each test. The table below provides insight the minimal (required), recommended, and optional information requested from the submitting lab.

**Minimal (Required)**
Test Tracking ID
GTR Accession (if updating or deleting test)
Action (update, delete, add, no change)
Laboratory test name
Purpose of the test
If licensed by NYS, NYS CLEP license number
If licensed by NYS, NYS CLEP expiration date
Test performance location(s)
Test performance location(s) comments
Method category
Test method
Analytical validity
Condition/phenotypes
Germline or somatic
Target category
Name of what is tested
If novel condition, conditions/phenotypes
If novel condition, indication type
**Recommended**
Test order code
Target population for test
Citations for target population
Test development
How to order (text description)
How to order (URL)
Test codes (URL)
Informed consent required?
Pre-test genetic counseling required?
Post-test genetic counseling required?
Specimen source
Specimen source URL
Testing strategy
Citations to support testing strategy
Test-specific contact policy
Test-specific contact person
Instruments used for test method
Confirmation of test results
Citations to support analytical validity
Clinical validity
Citations to support clinical validity
Assay limitations
Citations to support assay limitations
Clinical utility category
Is proficiency testing performed for this test?

Provider for proficiency testing
Description of proficiency testing method
Citations to support proficiency testing description
Description of internal test validation method
Citations to support internal test validation description
Major CAP category
CAP category
**Optional**
Laboratory test short name
Manufacturer’s test name
URL for the test
Search terms
Has there been FDA review?
If no FDA review, FDA category designation
If FDA review, item reviewed
If FDA review, FDA regulatory status
NYS CLEP status
LOINC codes
CPT codes
Who can order this test?
Test service
Test service order code
Test service comment
Test additional service
Test additional service order code
Test additional service comment
Platforms
Test procedure or protocol
Citations to support test procedure or protocol
Test comment
Variants
Clinical significance of variant
Citations to support the clinical significance of variant
Reference sequence: relevant exons
VUS: What is the protocol for interpreting a variation as a VUS?
VUS: What software is used to interpret novel variations?
VUS: What is the laboratory’s policy on reporting novel variations?
VUS: Are family members recruited without charge?
VUS: Comments about recruiting family members
VUS: Will the lab re-contact the ordering physician if variant interpretation changes?
VUS: Comments about re-contacting the ordering physician
VUS: Research performed after clinical testing is complete
CAP test list
If novel condition, lab displayed condition preferred name
If novel condition, suggest synonyms for condition
If novel condition, lab displayed acronym for the condition
If novel condition, suggest acronyms for condition
If novel condition, mode of inheritance for the condition
If novel condition, disease mechanism for the condition
If novel condition, prevalence (text description)

If novel condition, prevalence (URL)
If novel condition, citations to support prevalence
If novel condition, comments to GTR staff about condition

†The full submission form can be found on the GTR web page: https://www.ncbi.nlm.nih.gov/gtr/docs/fulltest
